# *MAT* heterozygosity and the second sterility barrier in the reproductive isolation of *Saccharomyces* species

**DOI:** 10.1007/s00294-020-01080-0

**Published:** 2020-04-30

**Authors:** Matthias Sipiczki, Zsuzsa Antunovics, Adrienne Szabo

**Affiliations:** Department of Genetics and Applied Microbiology, University of Debrecen, Debrecen, Hungary

**Keywords:** Interspecies hybridisation, Sterility barrier, Yeast, *MAT*, Mating, Sporulation

## Abstract

The genetic analysis of large numbers of *Saccharomyces cerevisiae* × *S. uvarum* (“cevarum”) and *S. kudriavzevii* × *S. uvarum* (“kudvarum”) hybrids in our previous studies revealed that these species are isolated by a postzygotic double-sterility barrier. We proposed a model in which the first barrier is due to the abruption of the meiotic process by the failure of the chromosomes of the subgenomes to pair (and recombine) in meiosis and the second barrier is assumed to be the result of the suppression of mating by allospecific *MAT* heterozygosity. While the former is analogous to the major mechanism of postzygotic reproductive isolation in plants and animals, the latter seems to be *Saccharomyces* specific. To bolster the assumed involvement of *MAT* in the second sterility barrier, we produced synthetic alloploid two-species cevarum and kudvarum hybrids with homo- and heterothallic backgrounds as well as three-species *S. cerevisiae* × *S. kudvarum* × *S. uvarum* (“cekudvarum”) hybrids by mass-mating and examined their *MAT* loci using species- and cassette-specific primer pairs. We found that the allospecific *MAT* heterozygosity repressed *MAT* switching and mating in the hybrids and in the viable but sterile spores produced by the cevarum hybrids that had increased (allotetraploid) genomes. The loss of heterozygosity by meiotic malsegregation of *MAT*-carrying chromosomes in the latter hybrids broke down the sterility barrier. The resulting spores nullisomic for the *S. uvarum* chromosome produced vegetative cells capable of *MAT* switching and conjugation, opening the way for GARMe (Genome Autoreduction in Meiosis), the process that leads to chimeric genomes.

## Introduction

Nature employs various ‘barriers’ to keep closely related species distinct from one another. One of these barriers is reproductive isolation that maintains the integrity of species over time by preventing genetic admixture (Coyne and Orr 2004). The reproductive isolation mechanisms can be prezygotic or postzygotic depending on whether they operate before or after fertilisation (Seehausen et al. 2014). The former prevents the formation of viable hybrid zygotes; while, the latter results in hybrids in which the incompatibility of the parental genomes causes aberrant development, low fitness, and/or sterility (Ouyang and Zhang 2013). Hybrid sterility is one of the earliest reproductive isolation mechanisms to evolve between two recently diverged species (Coyne and Orr 2004). The hybrids of such species are viable but incapable of sexual reproduction. Their sterility is mainly due to the failure of the chromosomes of the subgenomes to pair in meiosis (e.g. White 1978; Levin 2002; Li et al. 2009; Sigel 2016; Lavinscky et al. 2017; Forejt 1996) and/or to functional incompatibility of genes involved in gametogenesis (Johnson 2010). In the former case, the synteny and sequence differences prevent the (homeologous, allosyndetic) chromosomes of the subgenomes from efficient pairing in prophase-I of meiosis. This problem can be circumvented by genome duplication because within the duplicated subgenomes, each chromosome has a homologous (autosyndetic) partner to pair with, without interacting (allosyndetic pairing) with the chromosomes of the companion subgenome. As a result, each subgenome divides essentially autonomously and each gamete receives a copy of each chromosome of each subgenome. This process resulting in allodiploid gametes is referred to as autodiploidisation of the allotetraploid meiosis (Hutchinson et al. 1983). The allodiploid gametes can mate with other allodiploid gametes to produce allotetraploid progeny (Rieseberg and Willis 2007). If the subgenomes are functionally compatible, the allotetraploid hybrids (in contrast to the allodiploids) are capable of sexual propagation and can evolve into novel species distinct from the parental species. It is generally accepted that many plant species evolved in this way (by hybrid speciation) from interspecies hybrids (e.g. Soltis and Soltis 2009).

The species of the budding-yeast genus *Saccharomyces* are postzygotically isolated by a double-sterility barrier (for a recent review, see Sipiczki 2018). The *Saccharomyces* species form viable allodiploid hybrids in all combinations. The hybrid cells propagate asexually by mitotic divisions but cannot produce gametes (viable ascospores) by meiosis. Like in animal and plant allodiploid hybrids, the failure of viable gamete production (sporulation deficiency) in the *Saccharomyces* hybrids is also due to poor and promiscuous pairing between the chromosomes of the subgenomes. The inability of the allodiploid hybrids to produce viable ascospores is the first part of the double-sterility barrier. However, the spore viability problem can be circumvented by genome duplication. Like the allotetraploid plant hybrids, the allotetraploid *Saccharomyces* hybrids also produce viable gametes (ascospores) but, unlike the plant and animal allodiploid gametes, the allodiploid yeast ascospores cannot function as gametes because they cannot mate (fertilise). Thus, in the *Saccharomyces* hybrids, the restoration of spore viability does not imply that fertility is also restored. The mating incompetence of the allodiploid spores represents the second sterility barrier (Pfliegler et al. 2012) which has no equivalent in plants. This difference between the biological isolation of the plant species and the *Saccharomyces* species was proposed to be due to the different modes of the genetic determination of sexuality (Pfliegler et al. 2012). Being viable, the allodiploid spores germinate and produce clones of vegetatively propagating sterile cells (so-called spore clones or clones of “propagating gametes”) which is another difference from higher plants.

Our previous studies on “cevarum” (*S. cerevisiae* × *S. uvarum*) and “kudvarum” (*S. kudriavzevii* × *S. uvarum*) hybrids revealed that the allopolyploid hybrids can occasionally form fertile spores. These spores generate clones of cells which are able to mate with each other and the resulting “intraclonal F2 hybrids” also produce fertile spores. We noticed that these fertile spores had alloaneuploid genomes nullisomic for one or the other parental Chr III (Antunovics et al. 2005; Pfliegler et al. 2012; Karanyicz et al. 2017). As Chr III of *S. cerevisiae* and its counterparts in the other species carry the *MAT* locus, we proposed that the reactivation of the sexual processes (break-down of sterility barrier) is attributable to the loss of *MAT* heterozygosity (Pfliegler et al. 2012). All sterile spores had both parental types of Chr III; whereas, all fertile spores had only one type of Chr. III.

The “mating-type locus” *MAT* is the central regulator of the sexual processes (both gametogenesis/sporulation and fertilisation/mating) in *S. cerevisiae* (for reviews, see, e.g. Nasmyth 1982; Herskowitz 1988; Haber 2012; Thon et al. 2019). It has two idiomorphs (orthologous cassettes), *MATa* and *MATalpha*. In homothallic background (the *HO* gene is active), haploid cells can reversibly switch their mating types during vegetative propagation (by reversible replacement of the *MAT* cassettes in the *MAT* locus) and can conjugate with other haploid cells of opposite mating type but cannot sporulate. This is because both the *MATa* and the *MATalpha* alleles (cassettes) activate the mating-type switching machinery and the so-called haploid-specific genes whose activity is required for mating but both alleles repress the diploid-specific genes that are required for meiosis and sporulation. The mating of a *MATa* cell with a *MATalpha* cell results in a *MATa/MATalpha* zygote. The zygote produces vegetative cells of *MATa/MATalpha* genotype. In these cells, the proteins encoded by the different *MAT* cassettes interact to suppress mating-type switching and mating, but activate the diploid-specific genes that launch meiosis and sporulation in response to starvation signals. Thus, the haploid cells having single *MAT* loci (either *MATa* or *MATalpha*) are mating competent and sporulation incompetent; whereas, the diploid cells having both *MATa* and *MATalpha* cassettes are mating deficient and sporulation competent. Another difference between them is that the haploid cells can switch mating types; whereas, the *MATa/MATalpha* diploids cannot. As all *Saccharomyces* species have *MAT* loci which are interchangeable between the species (e.g. Pfliegler et al. 2012), the sexual processes are regulated much in the same way in the entire genus. Our model of the double-sterility barrier assumes that the allodiploid spores are sterile because they receive copies of different *MAT* cassettes from the subgenomes during the autodiploidised allotetraploid meiosis (Karanyicz et al. 2017) and the interactions of the cassettes block the mating process. The suppression of the mating activity which prevents the allodiploid spores from functioning as gametes is the second sterility barrier. The model also assumes that the subgenomes preserve the cassettes of the hybridising parental cells unchanged (no cassette switching takes place) during the propagation of the hybrid cells. The role of *MAT* in the sterility barrier may not be confined to *Saccharomyces*. *MAT* and *MTL* (*MAT*-like) loci were implicated in the sterility of alloploid *Zygosaccharomyces* strains isolated from natural substrates (Watanabe et al. 2017; Ortiz-Merino et al. 2017; Braun-Galleani et al. 2018; Bizzarri et al. 2019).

The proposed involvement of the *MAT* locus in the second sterility barrier relies upon the assumption that the *MAT* cassettes of the hybridising species remain active and functionally compatible with each other upon hybridisation. As this part of the model was largely hypothetical, we set out in this work to examine the *MAT* genotypes of hybrids and their viable spores. With this aim in view, we produced synthetic alloploid two-species cevarum (*S. cerevisiae* × *S. uvarum*) and kudvarum (*S. kudriavzevii* × *S. uvarum*) hybrids with homo- and heterothallic backgrounds as well as three-species cekudvarum (*S. cerevisiae* × *S. kudriavzevii* × *S. uvarum*) hybrids by crossing heterothallic and homothallic strains and examined their *MAT* loci. To be able to identify the *MATa* and *MATalpha* cassettes of each of the three species, we designed six pairs of species- and cassette-specific primers. Consistent with our model, the alloploid hybrids of the species and the sterile spores had complete alloploid karyotypes and stable *MAT* heterozygosity; whereas, the fertile spores were alloaneuploid, possessed only one parental Chr III, and formed clones in which the cells switched their mating types and mated with each other. The allotriploid cekudvarum hybrids were sterile and had three stable (non-switching) parental *MAT* cassettes.

## Materials and methods

### Strains and culture media

All strains used in this study are listed in Table 1. The strains involved in the tests of the *MAT*-specific primers and the parental strains used for hybridisation were maintained on YEA plates (yeast extract glucose agar) or in YEL broth (L stands for liquid). Mating tests were performed on YEA plates. Sporulation was tested on acetate SPA (sporulation agar). Hybrids were selected and maintained on MMA (minimal medium agar) or on MMA supplemented with uracil. The composition of the media was described in Sipiczki and Ferenczy (1977) and Antunovics et al. (2005).

**Table 1:** List of strains

Identification number	Strain	Genotype/phenotype	References
10-170	*Saccharomyces cerevisiae* X4005-11A	*MATa*^*Sc*^* ho*^*Sc*^* leu2*^*Sc*^	Antunovics et al. (2005)
10-512	*Saccharomyces uvarum* CBS 395	*MATa*^*Su*^	CBS
10-522	*Saccharomyces uvarum* m9	*MATa*^*Su*^*, MATalpha*^*Su*^* HO*^*Su*^* ura3*^*Su*^	Antunovics et al. (2005)
10-642	*Saccharomyces cerevisiae* ATCC 204508/S288c	*MATalpha*^*Sc*^* ho*^*Sc*^	ATCC
10-643	*Saccharomyces kudriavzevii* CBS 8840^T^	*MATa*^*Sk*^*, MATalpha*^*Sk*^* HO*	CBS
10-1650	*Saccharomyces uvarum* JRY9192 SSS110	*MATa*^*Su*^* ade2*^*Su*^* ura3*^*Su*^* ho*^*Su*^	Scannel et al. (2011)
10-1651	*Saccharomyces uvarum* JRY9193 SSS111	*MATalpha*^*Su*^* ade2*^*Su*^* ura3*^*Su*^* ho*^*Su*^	Scannel et al. (2011)
10-1652	*Saccharomyces kudriavzevii* FM1183 SSS410	*MATalpha*^*Sk*^* trp1*^*Sk*^* ura3*^*Sk*^* ho*^*Sk*^	Scannel et al. (2011)
10-1653	*Saccharomyces kudriavzevii* FM1193 SSS411	*MATa*^*Sk*^* trp1*^*Sk*^* ura3*^*Sk*^* ho*^*Sk*^	Scannel et al. (2011)
A2, A3, A27	Two-species cevarum hybrids produced with mass-mating of 10-170 and 10-522	*MATa*^*Sc*^*/MATalpha*^*Su*^* HO*^*Su*^*/ho*^*Sc*^* LEU2*^*Su*^*/leu2*^*Sc*^* URA3*^*Sc*^*/ura3*^*Su*^	This study
A3/1a, A3/1c	Spore clones of cevarun A3	Prototrophic	This study
A3/1b, A3/1d	Spore clones of cevarun A3	*leu2*^*Sc*^	This study
II/1, II/6	Two-species kudvarum hybrids produced with mass-mating of 10-1651 and 10-1653	*MATa*^*Sk*^*/MATalpha*^*Su*^* ho*^*Sk*^*/ho*^*Su*^* ADE2*^*Sk*^*/ade2*^*Su*^* trp1*^*Sk*^*/TRP1*^*Su*^* ura3*^*Sk*^*/ura3*^*Su*^	This study
II/6.1, II/6.2, II/6.3	Three-species cekudvarum hybrids produced with mass-mating of 10-170 and II/6	*MATa*^*Sc*^*/ MATa*^*Sk*^*/MATalpha*^*Su*^* ho*^*Sc*^*/ho*^*Sk*^*/ho*^*Su*^* ADE2*^*Sc*^*/ADE2*^*Sk*^*/ade2*^*Su*^* TRP1*^*Sc*^*/trp1*^*Sk*^*/TRP1*^*Su*^* URA3*^*Sc*^*/ura3*^*Sk*^*/ura3*^*Su*^* leu2*^*Sc*^*/LEU2*^*Sk*^*/LEU2*^*Su*^	This study

CBS: CBS-KNAW Collections, Westerdijk Fungal Biodiversity Institute, Utrecht, The Netherlands

ATCC: American Type Culture Collection, Manassas, VA 20108, USA

### Hybridisation

Two types of synthetic two-species hybrids were produced. The cevarum hybrids were constructed by mass-mating of 10-170 *S. cerevisiae* and 10-522 *S. uvarum* cultures grown on the sporulation medium SPA in YEL. The sporulation step was included in the procedure to increase the efficiency of mating and ensure that preferentially haploid × haploid mating occurs. The cells of the homothallic 10-522 *S. uvarum* strain are diploid and cannot mate due to their *MATa/MATalpha* heterozygosity. Its spores are haploid of either *a* or *alpha* mating type. The latter can mate with the haploid cells of 10-170 *S. cerevisiae* in the complete medium YEL (Antunovics et al. 2005; Pfliegler et al. 2012). To obtain kudvarum hybrids, exponential-phase 10-1651 *S. uvarum* and 10-1653 *S. kudriavzevii* cultures were mass-mated in YEL. Conjugation was monitored microscopically in both cases and samples containing zygotes were spread on MMA plates (for the identification of cevarum hybrids) or MMA plates supplemented with uracil (for the identification of kudvarum hybrids). To produce three-species cekudvarum hybrids, cells of exponential-phase 10-170 *S. cerevisiae* and II/6 kudvarum cultures grown in YEL were mass-mated in YEL. Their hybrids formed prototrophic colonies on MMA plates. Individual colonies (as products of individual zygotes) were isolated from the plates and stored at − 80 °C to preserve the homogeneity of the hybrid cell populations as much as possible. We considered this precaution necessary because the hybrid genomes can segregate during mitotic propagation of the cells (for a review, see Sipiczki, 2018). We used this culture as inoculum in all experiments instead of streaking out samples and selecting a colony which might already be a segregant. The hybrid nature of the isolates was verified by comparing their electrophoretic karyotypes with those of the parental strains as described previously (Antunovics et al. 2005) and by PCR–RFLP analysis of the *FUS1* gene (see in “Molecular methods”).

### Mating and sporulation tests and generation of spore clones

Mating activity was tested in exponential-phase mixed cultures. Equal volumes of overnight cultures (~ 5 × 10^6^ cells ml) of the strains grown in YEL were mixed, vortexed, centrifuged and then 10 µl of the wet pellet was dropped on YEA. After incubation at room temperature for 4–6 h, samples were taken from the mixed population of cells and examined microscopically. The testers of mating competences were *S. cerevisiae* 10-170 (*MATa*), *S. cerevisiae* 10-642 (*MATalpha*), *S. kudriavzevii* 10-1652 (*MATalpha*), *S. kudriavzevii* (*MATa*), *S. uvarum* 10-1650 (*MATa*) and *S. uvarum* 10-1651 (*MATalpha*).

Spore viability was examined by tetrad analysis. Cells of the hybrid to be tested were inoculated onto SPA plates and incubated for 5 days at room temperature. Four-spore asci were pulled out from samples of the sporulating culture transferred onto YEA plates, dissected by micromanipulation and their spores were separated. The viable spores formed colonies on the medium. As the colonies were formed by single spores, we considered them spore clones (clones of vegetative descendants of spores). The clones to be further examined were isolated and preserved at – 70 °C to prevent genomic changes by GARMi (Sipiczki 2018).

### Molecular methods

For the amplification of nuclear sequences, genomic DNA was isolated from 50-ml overnight cultures grown in YEL at 25 °C as described in Antunovics et al. (2005). Segments of the *FUS1* genes were amplified with two primer pairs. FUS1F (ACCGCAGCATATACTGACACC) and FUS1R (ACTTTTTCACCCAGCGAGAT) amplified a 870-bp-long fragment from *FUS1*^*Sc*^ and *FUS1*^*Su*^ but did not recognise *FUS1*^*Sk*^; whereas, FUS1kF (CGACAACAACTGTGATGACGAC) and FUS1kR (TGAAATATGTAGAACCTCTCAAGAACC) produced a 792-bp-long fragment from *FUS1*^*Sk*^. To distinguish the *FUS1* markers of the species, the amplicons were digested with *Taq*I that generated unique restriction patterns for each species (*S. cerevisiae*: 342, 266, 262; *S. kudriavzevii*: 473, 276, 43; *S. uvarum*: 371, 336, 163). The *MAT* cassettes were amplified by primer pairs suitable for the specific amplification of regions of the three types of *MATa* and the three types of *MATalpha* cassettes. The sequences of the primers and the length of the amplicons are shown in Table 2. PCR reactions were performed with the following programme: 95 °C for 5 min, 30 × (94 °C for 1 min, *T*_m_ × °C for 1 min), 72 °C for 5 min. *T*_m_ was set to 47 °C for the amplification of the *MATa* segments of all species and *MATalpha*^*Su*^, 50 °C for the amplification of the rest of the *MAT* sequences, and 54 °C for *FUS1*. As certain *MAT* amplicons did not differ significantly in size, we did not apply multiplex PCR. All *MAT* amplifications were carried out with single primer pairs. The amplified DNA fragments were examined by electrophoresis in agarose gel. Electrophoretic karyotyping was performed with the Bio-Rad CHEF Mapper system as described previously (Antunovics et al. 2005).

**Table 2:** Primers used for the identification of the *MAT* cassettes of the species

Species	Primer	Sequence	Amplicon size (bp)	
*S. cerevisiae*	MATa^Sc^	F: CCACATTAAAAAAGAGAAGAGC	MATa^Sc^–Scout:	509
	MATalpha^Sc^	F: TAAAATCCAAATTCACAGGATAGCGTCT	MATalpha^Sc^- Scout:	672
	Scout	R: TATGGTTAAGATAAGAACAAAGAATG		
*S. kudriavzevii*	MATa^Sk^	F: GTATGAAAAATCAAGCTAA	MATa^Sk^- Skout:	309
	MATalpha^Sk^	F: GTAATGGCATAGTGAAACGAATAAGT	MATalpha^Sk^- Skout:	657
	Skout	R: GTAAATACCTCAAAGGAATTATCA		
*S. uvarum*	MATa^Su^	F: CAACGTGAATCAATCCTAA	MATa^Su^- Suout:	439
	MATalpha^Su^	F: TCGAGAAAAGCATCAATAACAC	MATalpha^Su^- Suout:	603
	Suout	R: TCACCAAATACGAAAAGTAA		

## Results

### Hybridisation

**Fig. 1:**
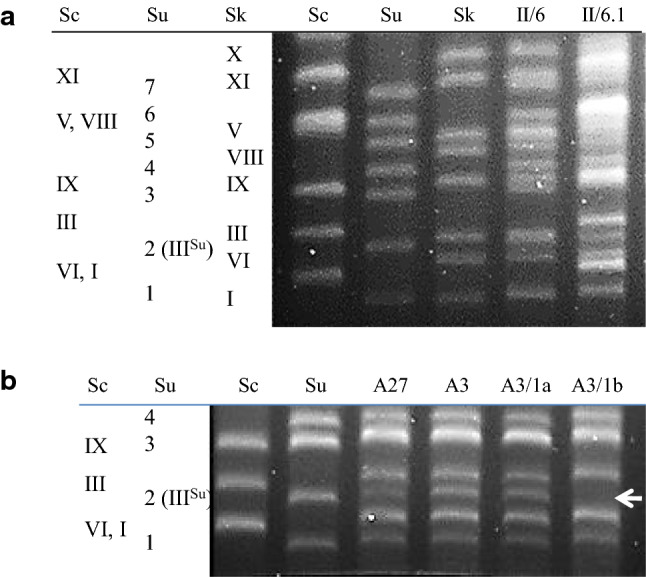
Karyotypes of parental strains, hybrids and spore clones. **a** Karyotypes of a two-species kudvarum hybrid, a three-species cekudvarum hybrid and the parental strains. Only the regions of smaller chromosomes are shown in which individual bands can be distinguished in the cekudvarum karyotype. **b** Karyotypes of two-species cevarum hybrids and spore clones. Only the regions containing the bands of the *MAT*-carrying chromosomes are shown. The columns on the left sides of the gel photographs show the conventional numbering of chromosomes in the *S. cerevisiae*, *S. uvarum* and *S. kudriavzevii* genomes. The *S. uvarum* chromosomes are numbered according to Nguyen et al (2000). Sc: *S. cerevisiae* 10-170; Sk: *S. kudriavzevii* 10-1653; Su: *S. uvarum* 10-522; II/6: kudvarum hybrid; II/6.1: cekudvarum hybrid; A27 and A3: cevarum hybrids, A3/1a: sterile spore clone of A3; A3/1b: fertile spore clone of A3. Arrowhead marks the position of the missing Chr III^Su^ (Chr 2) in the fertile spore clone A3/1b

For the examination of the involvement of the *MAT* alleles in the second sterility barrier, two-species cevarum and kudvarum hybrids and three-species cekudvarum hybrids were constructed. The hybrids were subjected to karyotyping and mating tests with the heterothallic parental strains. All hybrids had hybrid karyotypes (examples are shown in Fig. 1) and none of them conjugated (mated) with any of the testers. From these results, we inferred that the hybrids were alloploid and heterozygous at the *MAT* locus.

The cevarum hybrids obtained from mass-mating of the heterothallic 10-170 *S. cerevisiae* and the homothallic 10-522 *S. uvarum* strains had inactive *ho*^*Sc*^ and functional *HO*^*Su*^ genes. The mass-mating of the heterothallic 10-1653 *S. kudriavzevii* and 10-1651 *S. uvarum* cells resulted in kudvarum hybrids having inactive *ho* genes in both subgenomes. For further studies, we selected three *HO*^*Su*^*/ho*^*Sc*^ cevarum hybrids (A2, A3 and A27) and two *ho*^*Sk*^*/ho*^*Su*^ kudvarum (II/1 and II/6) hybrids.

As both 10-1653 and 10-1651 were *ura3*, the kudvarum hybrids were auxotrophic for uracil. To produce three-species cekudvarum hybrids with the method described in Sipiczki (2019), one of them (II/6) was mass-mated with the heterothallic leucine-auxotrophic *S. cerevisiae* strain 10-170. Although the kudvarum parent proved to be inactive in the mating tests, we presumed that their mating block was not absolute and mating-competent allodiploid cells might occasionally be produced during vegetative propagation. This assumption was based on the observations that heterozygous *MATa/MATalpha S. cerevisiae* cells converted at very low frequency to homozygous cells capable of mating (“rare mating”) by mitotic gene conversion (Gunge and Nakatomi 1972). As expected, a few prototrophic colonies appeared on the selective medium when larger samples of the mass-mated mixed population were spread on the plates. The karyotypes of these colonies had bands characteristic of the *S. cerevisiae* parent in addition to the kudvarum bands (Fig. 1a) and their cells did not conjugate with cells of the parental strains. To reinforce the karyotyping results, we also examined the *FUS1* genes located on Chr III (between *MAT* and the centromere) in the genomes of the parental species (Scannel et al. 2011). The PCR–RFLP analysis detected all the three orthologues in the prototrophic colonies. The prototrophic phenotype, the presence of characteristic chromosomal bands of three parental strains and the presence of all parental versions of the *FUS1* marker implied that these colonies were allotriploid cekudvarum hybrids. The *FUS1* genotype and the lack of mating competence further indicated that they had heterozygous *MAT* genotypes.

### Hybrid ploidy

Numerous previous studies found that allodiploid *Saccharomyces* hybrids possessing single sets of parental chromosomes were either defective in sporulation or produced viable spores at extremely low frequencies; whereas, the spores of the allopolyploids that had duplicated sets of chromosomes in the subgenomes were viable (for a review, see Sipiczki 2018). Thus, allodiploid hybrids can be distinguished from allopolyploid hybrids by testing their spores for viability. We found that the cevarum hybrid A27 formed no spores; whereas, A2 and A3 and the kudvarum hybrids II/1 and II/6 formed asci when being cultivated on the sporulation medium SPA. To examine the viability of the spores of the latter hybrids, 10 four-spored asci were dissected with micromanipulation from each sporulating culture and their spores were separated on the complete medium YEA. None of the spores of the kudvarum hybrids formed colonies; whereas, high proportions (32/40 and 37/40, respectively) of the spores of the cevarum hybrids A2 and A3 proved viable. From these results, we inferred that the cevarum hybrid A27 and the kudvarum hybrids were allodiploids; whereas, the ploidy of A2 and A3 was higher. The high level of spore viability indicates that the latter hybrids had allotetraploid genomes because triploids produce viable spores at much lower rates even when they have autoploid genomes (e.g. St Charles et al. 2010). Allotetraploids can arise from allodiploids by spontaneous genome duplication. Recent studies demonstrated that genome duplication by endoreduplication is a fairly common event in *Saccharomyces* (Harari et al. 2018). Besides, all spores of A2 and the majority of the spores of A3 formed prototrophic colonies. As the hybrids were heterozygous *LEU2*^*Su*^*/leu2*^*Sc*^* URA3*^*Sc*^*/ura3*^*Su*^*,* their prototrophy indicated that they were heterozygous allodiploid products of the tetraploid meiosis. A shown previously, *Saccharomyces* allotetraploids form allodiploid spores due to the autodiploidisation of the allotetraploid meiosis (Karanyicz et al. 2017). None of the spores of the three-species cekudvarum hybrid II/6.1 formed colonies, suggesting that they had single sets of parental chromosomes.

### Meiotic segregation

**Fig. 2:**
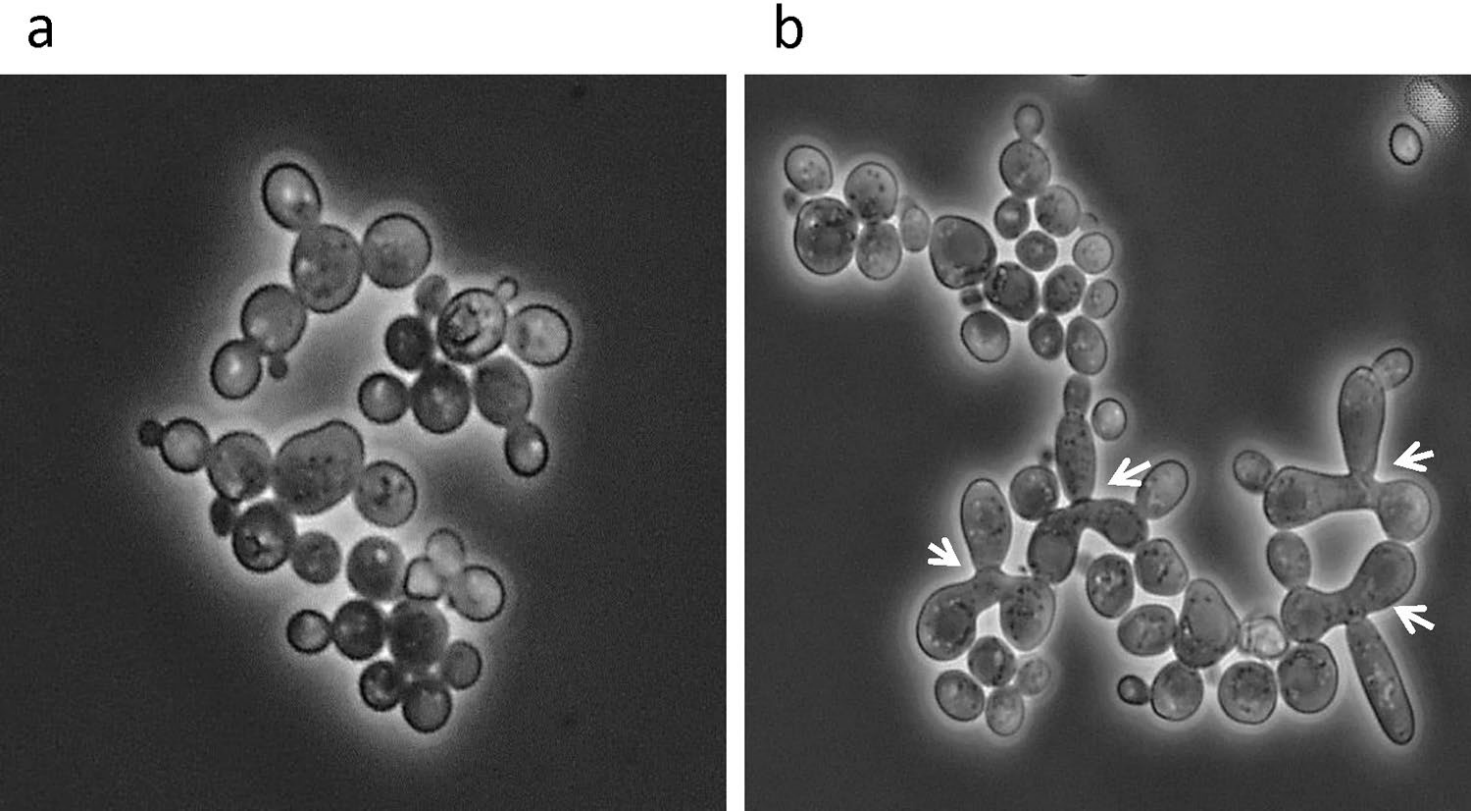
Microscopic images of cultures tested for mating. **a** Cells of the sterile spore clone A3/1a. **b** Cells of the fertile spore clone A3/1b. Arrowheads point to germinating zygotes

However, not all spores of the hybrid A3 were prototrophs. In one of the tetrads of A3, two spores (A3/1b and A3/1d) formed colonies auxotrophic for lucine. As A3 had *LEU2*^*Su*^*/leu2*^*Sc*^ heterozygous genotype, these spores must have been aneuploid products of segregation bias against the *S. uvarum* chromosome carrying the wild-type *LEU2*^*Su*^ gene. Occasional loss of *LEU2*^*Su*^*/leu2*^*Sc*^ heterozygosity during cevarum allotetraploid meiosis was also observed in previous studies and found to correlate with the loss of Chr. III^Su^ that carries this gene, near its centromere (Antunovics et al. 2005; Pfliegler et al. 2012). Consistent with those observations, the karyotype of the leu^−^ spore clones of A3 lacked the band corresponding to the *S. uvarum* Chr III (Fig. 1). As the *MAT* locus is also located on this chromosome, with the loss of Chr. III^Su^, the spores also lost *MAT*^*Su*^. Thus the leu2^−^ spores were alloaneuploid, monosomic for Chr. III^Sc^ and hemizygous for *MATa*^*Sc*^. According to our model of the second sterility barrier, the loss of *MAT* heterozygosity relieves the mating block and restores mating competence and mating-type switching (Pfliegler et al. 2012). To verify the effect of the loss of *MAT* heterozygosity on the mating activity, we tested a leu^+^ (A3/1a) and a leu^−^ (A3/1b) spore clone for conjugation with the parental strains. The cells of the prototrophic clones conjugated neither with each other nor with the testers; whereas, the cells of the leu^−^ clones formed zygotes both within the clones (Fig. 2) and with both testers. This difference corroborates the notion that the loss of *MAT* heterozygosity breaks down the second sterility barrier and makes mating and mating-type switching possible.

### Stable allospecific *MAT* heterozygosity in the hybrids

**Fig. 3:**
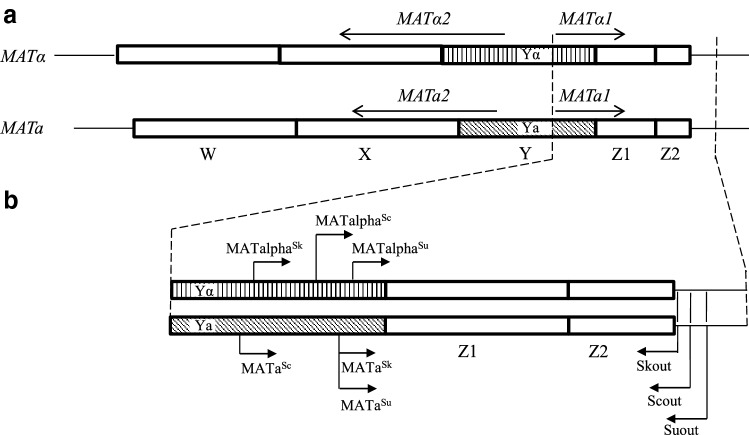
Selection of binding segments for primers that can be used to amplify species-specific sections of the *MAT* loci. **a** Structures of the *MATalpha* and *MATa* cassettes of *S. cerevisiae* distinguished by their Ya or Yalpha regions differing from each other both in length and in nucleotide sequence. W, X, Z1 and Z2 are conserved regions present both in the *MATalpha* and *MATa* cassettes (Haber 1998). **b** Locations of primers used for the amplification of species-specific segments of the *MAT* alleles

**Fig. 4:**
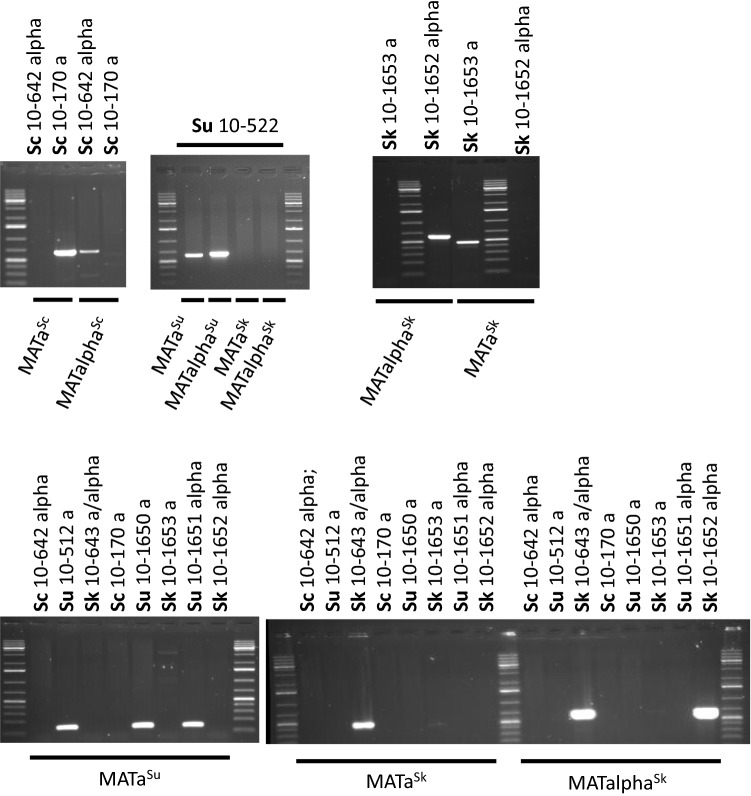
Verification of the specificity of primers. Amplification of *MAT* segments of homo- and heterothallic strains of *S. cerevisiae* (10-170, 10-642), *S. kudriavzevii* (10-643, 10-1652, 10-1653) and *S. uvarum* (10-512, 10-522, 10-1650, 10-1651) with primer pairs specific for *MATa*^*Sc*^, *MATalpha*^*Sc*^, *MATa*^*Sk*^, *MATalpha*^*Sk*^, *MATa*^*Su*^ and *MATalpha*^*Su*^. Sc: *S. cerevisiae*; Sk: *S. kudriavzevii*; Su: *S. uvarum*

Since hybridisation took place between different species, the zygotes and the hybrid clones formed by them had allospecific combinations of *MAT* cassettes. To examine the postzygotic fate of the cassettes, we examined the MAT loci of the hybrids. Although the cassettes have conserved structures, the species differ in the sequences of certain segments. The primers proposed by Huxley et al. (1999) and widely used for the detection of the *MATa* and *MATα* cassettes in *S. cerevisiae* and the primers used by us in a previous study to amplify the entire *MAT* locus of *S. cerevisiae* (Pfliegler et al. 2012) are complementary to segments conserved in the three species involved in this study. Therefore, we set out to design primers suitable for the specific identification of the cassettes of the three species (Fig. 3 and Table 2). In each pair, the forward primer is complementary to a segment of the Ya or Yalpha region variable in the species; while, the reverse primer hybridises to an external chromosomal sequence adjacent to the Z2 region which is also different in each species. By choosing sequences located outside of the cassettes for reverse primers, we could prevent amplification from the silent *HMR* and *HML* cassettes. The amplification tests proved that each pair is species- and cassette specific (Fig. 4 and Table 2).

Using the species- and cassette-specific primers, PCR reactions were performed with genomic DNAs extracted from the hybrids. From the cevarum hybrids, *MATa*^*Sc*^ and *MATalpha*^*Su*^ could be amplified; whereas, the *MATalpha*^*Sc*^ and *MATa*^*Su*^ primers generated no bands. The lack of the latter cassettes implies that these hybrids arose from conjugation of *MATa S. cerevisiae* cells with *MATalpha S. uvarum* spores and no cassette replacement (mating-type switching) took place during the vegetative propagation of the hybrid cells in spite of the presence of the wild-type *HO*^*Su*^ gene in their genomes. In the kudvarum hybrids II/1 and II/6, the PCR reactions detected *MATa*^*Sk*^ and *MATalpha*^*Su*^ cassettes which was consistent with their *ho*^*Sk*^*/ho*^*Su*^ genotype. The cekudvarum hybrid produced by “rare mating” of the *MATa*^*Sk*^*/MATalpha*^*Su*^* ho*^*Sk*^*/ho*^*Su*^ kudvarum hybrid with the *MATa*^*Sc*^* ho*^*Sc*^* S. cerevisiae* strain possessed all parental *MAT* cassettes. No cassette replacement (*MAT* switching) was detected in this hybrid either (Table 3).

**Table 3:** Amplification of *MAT* cassettes

Strain	Amplification with specific primer pairs					
	*S. cerevisiae*		*S. kudriavzevii*		*S. uvarum*	
	a	alpha	a	alpha	a	alpha
	MATa^Sc^ Scout	MATalpha^Sc^ Scout	MATa^Sk^ Skout	MATalpha^Sk^ Skout	MATa^Su^ Suout	MATalpha^Su^ Suout
*S. cerevisiae* 10-170 a	+	−	−	−	−	−
*S. cerevisiae* 10-642 alpha	−	+	−	−	−	−
*S. kudriavzevii* 10-643 a/alpha	−	−	+	+	−	−
*S. kudriavzevii* 10-1652 alpha	−	−	−	+	−	−
*S. kudriavzevii* 10-1653 a	−	−	+	−	−	−
*S. uvarum* 10-512 a	−	−	−	−	+	−
*S. uvarum* 10-522 a/alpha	−	−	−	−	+	+
*S. uvarum* 10-1651 alpha	−	−	−	−	−	+
Cevarum hybrids A2, A3 and A27	+	−	−	−	−	+
Cevarum spore clones A3/1a and A3/1c	+	−	−	−	−	+
Cevarum spore clones A3/1b and A3/1d	+	+	−	−	−	−
Kudvarum hybrids II/1 and II/6	−	−	+	−	−	+
Cekudvarum hybrid II/6.1	+	−	+	−	−	+

### Reactivated *MAT* switching upon the loss of allospecific *MAT* heterozygosity

**Fig. 5:**
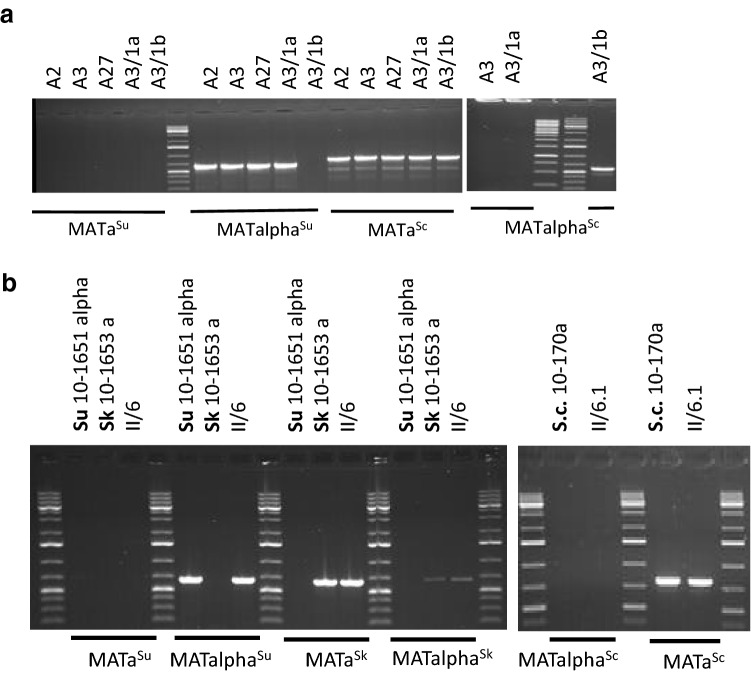
Examination of the *MAT* genotypes of hybrids and spore clones with species-specific primers. **a** *HO/ho* cevarum hybrids (A2, A3 and A27), the sterile spore clone A3/1a and the fertile spore clone A3/1b. All hybrids and the sterile spore clone have *MATa*^*Sc*^*/MATalpha*^*Su*^ genotypes. The fertile spore clone lacks *MATalpha*^*Su*^ but has *MATalpha*^*Sc*^. **b** The *ho/ho* kudvarum hybrid II/6 and the *ho/ho/ho* cekudvarum hybrid II/6.1. The kudvarum hybrid has *MATa*^*Sk*^*/MATalpha*^*Su*^ genotype whereas the cekudvarum hybrid also has a *MATa*^*Sc*^ cassette

The ability of the cells of the alloaneuploid spore clones A3/1b and A3/1d to produce zygotes within the clones and with both mating-type testers indicated that these clones were heterogeneous in terms of the mating activities of their cells. As the cells had *MAT*-carrying chromosome(s) only in the *S. cerevisiae* subgenome, heterogeneity could only be attributed to cassette replacements (MAT switching) in the *S. cerevisiae MAT* locus. To bolster this conclusion with experimental results, we amplified *MAT* cassettes from the cells of these clones (Fig. 5). As expected, we detected both *S. cerevisiae*-type cassettes (*MATa*^*Sc*^ and *MATalpha*^*Sc*^) but neither S*. uvarum*-type cassette. To verify that the cells of these clones were *MATa*^*Sc*^/ *MATalpha*^*Sc*^ heterozygotes, samples of their cultures were plated out on YEA and individual colonies were isolated. Three colonies for each clone were tested for *MAT*. All were heterozygotes. In contrast, the non-conjugating allodiploid spore clones A3/1a, A3/1c preserved the *MATa*^*Sc*^*/MATalpha*^*Su*^ genotype of the A3 hybrid. Table 4 summarises all the relevant properties of the spore clones of the cevarum tetrad A3/1. We also tested the *MAT* genotype in four prototrophic and one additional leu^−^ clone randomly selected from other tetrads. The prototrophs had allospecific *MAT* heterozygosity; whereas, the leu^−^ clone was heterozygous for the *S. cerevisiae MAT* cassettes.

**Table 4:** Properties of the spore clones of the cevarum tetrad A3/1

Spore clone	Phenotype	ChrIII	*HO*	*MAT*	Viable spores
A3/1a	Prototrophic	ChrIII^Sc^, ChrIII^Su^	*HO*^*Su*^*/ho*^*Sc*^	*MATa*^*Sc*^*/MATalpha*^*Su*^	−
A3/1b	Leu^−^	ChrIII^Sc^	*HO*^*Su*^*/ho*^*Sc*^	*MATa*^*Sc*^*/MATalpha*^*Sc*^	+
A3/1c	Prototrophic	ChrIII^Sc^, ChrIII^Su^	*HO*^*Su*^*/ho*^*Sc*^	*MATa*^*Sc*^*/MATalpha*^*Su*^	−
A3/1d	Leu^−^	ChrIII^Sc^	*HO*^*Su*^*/ho*^*Sc*^	*MATa*^*Sc*^*/MATalpha*^*Sc*^	+

## Discussion

In previous analyses of alloploid *Saccharomyces* hybrids, we noticed that the species of the genus are reproductively isolated by a pair of postzygotic sterility barriers which ensure that neither the allodiploid (first barrier) nor the allopolyploid (second barrier) hybrids produce functional gametes of euploid genomes (for a review, see Sipiczki 2018). We proposed a model in which the first barrier is due to the abruption of the meiotic process by the failure of the chromosomes of the subgenomes to pair (and recombine) in the prophase-I of meiosis and the second barrier is attributed to *MAT* heterozygosity. While the former is analogous to the major mechanism underlying the reproductive isolation in plants and animals (Sigel 2016; Lavinscky et al. 2017; Forejt 1996), the latter seems to be *Saccharomyces* specific. In the model, the *MAT* system contributes to the sterility by the interactions of the *MATa* and *MATalpha* loci of the subgenomes that repress the activity of the mating-specific genes (required for fertilisation) and *MAT* switching (reciprocal cassette replacements in the *MAT* loci). The current study contributes to the experimental validation of the model by investigating the *MAT* loci of interspecies hybrids and their meiotic derivatives.

The results of the analysis of the *MAT* loci of the two- and three-species hybrids and the viable but sterile allodiploid spores of an allotetraploid two-species hybrid carried out in this study corroborate the proposed interaction of the allospecific *MAT* cassettes to silence the mating functions. Neither the two-species cevarum (*MATa*^*Sc*^*/MATalpha*^*Su*^*)* and kudvarum (*MATa*^*Sk*^*/MATalpha*^*Su*^*)* hybrids nor the three-species cekudvarum (*MATa*^*Sc*^*/ MATa*^*Sk*^*/MATalpha*^*Su*^*)* hybrids conjugated with the tester heterothallic strains. The non-mating allodiploid gametes (ascospores) of the cevarum hybrid producing viable spores were also *MATa*^*Sc*^*/MATalpha*^*Su*^*.* No *MATa*^*Su*^ and *MATalpha*^*Su*^ cassettes were detected in their cells. The correlation of the inability of the alloploid cells of these spore clones to mate and the allospecific combinations of *MATa* and *MATalpha* cassettes in their genomes bolsters the notion that *MAT* heterozygosity silences their mating-specific genes and, hence, prevents them from acting as gametes.

Our double-sterility model further assumes that the interaction of the allospecific *MAT* cassettes also represses *MAT* switching. Consistent with this assumption, we found that both the two-species allodiploid and the three-species allotriploid hybrids preserved the cassettes of their parental strains unchanged. The parental cassettes did not change in the *MAT* loci of the sporulating tetraploid hybrid and its non-mating allodiploid spore clones either.

It was also proposed in the model that the allospecific *MATa* and *MATalpha* cassettes can cooperatively launch meiosis. Consistent with this proposal, two of the cevarum hybrids and the kudvarum hybrids formed spores. However, only the cevarum spores were viable. As the meiotic pairing and segregation of chromosomes require two sets of chromosomes in both subgenomes (autodiploidisation of meiosis, Karanyicz et al. 2017), these hybrids must have undergone genome duplication. We also observed frequent genome-size increase in our previous studies of cevarum and kudvarum hybrids (Pfliegler et al. 2012; Karanyicz et al. 2017). The alloploid karyotypes of their spores indicated that these hybrids had at least allotetraploid genomes. As neither subgenome had two different *MAT* loci, the meiosis was launched in these hybrids by the interaction of their complementary *MAT* loci (*MATa*^*Sc*^ and *MATalpha*^*Su*^). This interaction can also be functional in allodiploids, and allotriploids as demonstrated by the sporulation proficiency of the kudvarum hybrids II/1 and II/6 as well as the cekudvarum hybrids but cannot result in viable spores because of the abruption of the meiotic process by the lack of homologous partners for chromosome pairing within the subgenomes.

These results corroborate the tenet of the double-sterility barrier model that the second sterility barrier is attributable to allospecific *MAT* heterozygosity causing mating (fertilisation) incompetence. In the same time, this heterozygosity can launch meiosis which, however, gets stuck because of the failure of chromosome pairing. In principle, *MAT* heterozygosity can also confer spore sterility in autotetraploids. Even if it happens, it affects only a small fraction of spores (Pomper et al. 1954) because in an autotetraploid nucleus each chromosome can freely pair with any of its three homologues regardless of their origin.

Further proof bolstering the role of the *MAT* loci in the second sterility barrier is provided by the cevarum spore clones that lack Chr III^Su^. By losing this chromosome, the spore also loses the *MAT* locus of the *S. uvarum* subgenome. We found that these spores nullisomic for Chr III^Su^ formed clones of vegetative cells capable of mating with each other within the clones. Their mating competence implies that the loss of *MAT* heterozygosity relieves the switching machinery and the mating programme from repression and, thus, inactivates the second sterility barrier. In accordance with this, we also detected the *MATalpha*^*S*c^ cassette in the cultures of these clones. Obviously, *MAT* switching took place in the *S. cerevisiae* chromosome despite the absence of an active *HO*^*Sc*^ gene. This finding indicates that the wild-type *HO*^*Su*^ gene of the *S. uvarum* subgenome can functionally substitute its *S. cerevisiae* orthologue. Thus, not only the *MAT* cassettes but also the *HO* genes are interchangeable between *S. cerevisiae* and *S. uvarum* in spite of the difference between their sequences (de Melo Pereira et al. 2010). Due to the reactivation of the mating competence and the mating-type switching mechanisms, the cells of the leu^−^ spore clones can mate with each other and form zygotes. As they are nullisomic for Chr III^Su^, the resulting zygotes and their vegetative progeny will also be nullisomic for this chromosome but disomic for Chr III^Sc^ and heterozygous for the *S. cerevisiae MAT* cassettes (*MATa*^*Sc*^*/MATalpha*^*Sc*^). The *MAT* heterozygosity prevents them from further mating but allows sporulation because the a1p/alpha2p heterodimer protein complex activates *IME1* under starvation conditions that codes for a central regulator of many meiotic genes (for a review of the regulation of meiosis in *S. cerevisiae*, see Mitchell 1994).

**Fig. 6:**
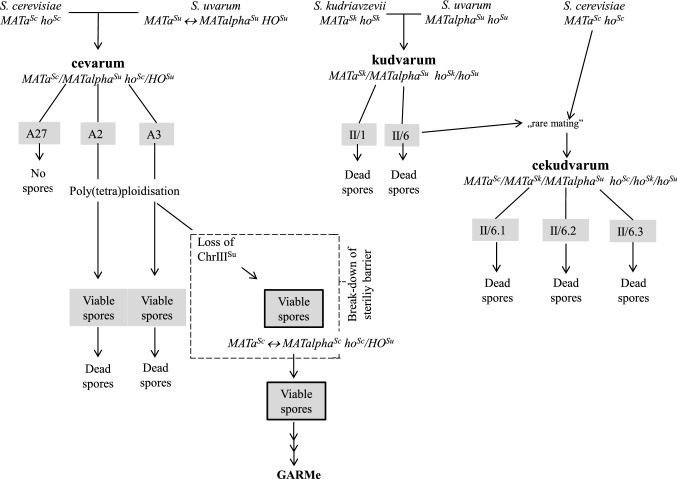
A general scheme showing the experimental strategy and the detected correlation between *MAT* genotypes, alloploid sterility and the break-down of the sterility barrier. Hybrids and viable spore clones are grey. ↔ : *MAT* switching. *GARMe* genome autoreduction by meiosis (Karanyicz et al. 2017)

The break-down of the second sterility barrier represents an escape route from the reproductive isolation (Pfliegler et al. 2012; Karanyicz et al. 2017). The cells of the spore clones nullisomic for one or the other parental Chr III can mate with each other and with mating-competent cells of other strains. In the former case, they form zygotes whose vegetative progeny produce fertile gametes which then generate additional generations of fertile zygotes. However, the loss of Chr. III destabilises the genome. Additional chromosomes can be lost during meiotic divisions in the successive generations of spore clones. This gradual genome reduction is referred to as GARMe (Karanyicz et al. 2017). Figure 6 depicts the correlation between the sexual behaviour and the *MAT* genotype in the alloploid hybrids, their sterile and fertile spores (gametes).

Recently *MAT* genes have been implicated in the evolution and sterility of certain natural *Zygosaccharomyces* strains whose genome structures indicated hybrid origin (Watanabe et al. 2017; Ortiz-Merino et al. 2017; Braun-Galleani et al. 2018; Bizzarri et al. 2019). These strains have complex and diverse repertoires of *MAT* and *MAT*-like (*MTL*) idiomorphs inherited from the partners of the ancient hybridisation events and modified later by intragenomic chimerisation events. Some of them were found to participate in the regulation of the sexual processes in ways somewhat different from the situation in *Saccharomyces*. For examples, the sterile allodiploid strain ATCC42981 is of *MATa/MATalpha* genotype but the deletion of the *MATalpha* locus did not restore fertility probably because of incomplete silencing at the chimeric *HMLalpha* cassette (Bizzarri et al. 2019). In *S. cerevisiae*, the *HMR* and *HML* loci contain completely silent *MATa* and *MATalpha* cassettes (reviewed in Haber 2012). In contrast to ATCC42981, the fertile natural interspecies hybrid ATCC60483 is assumed to have regained fertility as a consequence of irreparable damage to one of the two homeologous *MAT* loci that must have occurred some time ago during the evolution of the strain (Ortiz-Merino et al. 2017). The *MAT* genotypes of the natural *Zygosaccharomyces* hybrids indicate that a mechanism similar to the second sterility barrier of *Saccharomyces* species may also exist in *Zygosaccharomyces.* Genetic analysis of synthetic alloploid *Zygosaccharomyces* hybrids will reveal to what extent the mechanisms underlying the reproductive isolation of species are similar in these genera.

## Data Availability

All data generated or analysed during this study are included in this published article.
